# The role of innovative modeling and imaging techniques in improving outcomes in patients with LVAD

**DOI:** 10.3389/fcvm.2023.1248300

**Published:** 2023-08-24

**Authors:** Shannon I. Wilson, Katelyn E. Ingram, Albert Oh, Michael R. Moreno, Mahwash Kassi

**Affiliations:** ^1^ Department of Biomedical Engineering, Texas A&M University, College Station, TX, United States; ^2^ DeBakey Heart and Vascular- Heart Center Research, Houston Methodist Research Institute, Houston, TX, United States; ^3^ School of Engineering Medicine, Texas A&M University, Houston, TX, United States; ^4^ J. Mike Walker ‘66 Department of Mechanical Engineering, Texas A&M University, College Station, TX, United States; ^5^ Cardiology, DeBakey Heart and Vascular, Houston Methodist Hospital, Houston, TX, United States

**Keywords:** LVAD, imaging, HRAEs, hemodynamics, outcomes

## Abstract

Heart failure remains a significant cause of mortality in the United States and around the world. While organ transplantation is acknowledged as the gold standard treatment for end stage heart failure, supply is limited, and many patients are treated with left ventricular assist devices (LVADs). LVADs extend and improve patients' lives, but they are not without their own complications, particularly the hemocompatibility related adverse events (HRAE) including stroke, bleeding and pump thrombosis. Mainstream imaging techniques currently in use to assess appropriate device function and troubleshoot complications, such as echocardiography and cardiac computed tomography, provide some insight but do not provide a holistic understanding of pump induced flow alterations that leads to HRAEs. In contrast, there are technologies restricted to the benchtop—such as computational fluid dynamics and mock circulatory loops paired with methods like particle image velocimetry—that can assess flow metrics but have not been optimized for clinical care. In this review, we outline the potential role and current limitations of converging available technologies to produce novel imaging techniques, and the potential utility in evaluating hemodynamic flow to determine whether LVAD patients may be at higher risk of HRAEs. This addition to diagnostic and monitoring capabilities could improve prevention and treatment of LVAD-induced complications in heart failure patients.

## 1. Introduction

Heart failure remains a top public health concern, with significant morbidity and mortality especially among the aging population (1, 2). Cardiac transplantation is generally considered the gold standard for end-stage heart failure patients that are refractory to medical therapy however, it is not a viable option for patients who are not candidates for transplantation. In the modern medical world, LVADs have become an option for bridge-to-transplant, bridge-to-recovery, and destination therapy in the treatment of heart failure. Consequently, the monitoring and management of HRAEs associated with LVADs are primary concerns for cardiologists and cardiothoracic surgeons taking care of these patients. Currently, imaging technologies such as echocardiography and invasive hemodynamic monitoring are used to monitor these patients.

Today, Abbott's HeartMate 3 devices are the only approved LVADs in the United States (Abbott Laboratories, Abbott Park, IL, USA). The MOMENTUM 3 trial showed that th HeartMate 3 has a superior hemocompatibility profile compared to the older generation HeartMate II device, and has shown a reduction in HRAEs at 6 month, 2 year, and 5 year post-implantation intervals (3–5). Furthermore, the 5-year survival has significantly improved (up to 60%) with the HM3 device. However, HRAEs are still of significant consideration motivating continued pursuit of innovation both for LVAD design evolution and more sophisticated techniques for monitoring risk of negative outcomes (6). These pursuits take place on the benchtop, where academic and exploratory research utilize experimental and simulation based approaches, as well as in the clinic, where medical professionals work to push the limits of gold standard technologies to improve patient care. This review aims to bring together current standards of clinical care in monitoring LVAD hemodynamic status and standard approaches for benchtop investigations of LVAD design and performance, as well as highlight novel developments in both contexts that may directly contribute to the future gold standards of care. We hope readers can utilize this article as a tool in deciding which LVAD imaging technologies to use as well as to find encouragement to develop new monitoring technologies for bedside use through collaborative better technologies for LVAD development, implantation, and monitoring. Such translational efforts between medical professionals and medical device innovators can improve outcomes for end-stage heart failure patients.

## 2. Clinical imaging, monitoring and evaluation

Several imaging and monitoring technologies are used to evaluate LVAD performance and patient status at the bedside. While many of these are standard, their limits are being pushed to discover new techniques and uses. These approaches are summarized in Table 1.

**Table 1:** Clinical imaging approaches.

Method	Use cases	Primary limitations
Transthoracic echocardiography (TTE)	Serial evaluation of LVAD after implantation	Device artifact, 2-D planar imaging, narrow field of view, poor acoustic windows (7)
	Evaluation of anatomy and function of the heart	
Transesophageal echocardiography (TEE)	Rapid and accurate visualization of cardiac dysfunction especially useful in the setting of LVADs	Device artifact, 2-D planar imaging, narrow field of view, patient needs to be sedated which may be contraindicated in some cases.
	TEE offers a more proximal acoustic window by visualization through the esophagus	
Transhepatic echocardiography (THE)	Similar to transthoracic echocardiography in that it is completely non-invasive	Similar limitations to TTE.
	Has utility in cases of difficult acoustic windows using TTE.	
	Can be used if TTE window is not sufficient.	
Right heart catheterization	Assessment of risk to the right heart through analysis of pulmonary system pressure and pulmonary vascular resistance	Invasive
Hemodynamic ramp testing (8, 9)	Personalized LVAD speed adjustment based on patient hemodynamic profile measured by right heart catheterization and echocardiography	Involves invasive right heart catheterization and is subject to visualization limitations of echocardiography
Computed tomography (CT)	Whole scale 3-D imaging of heart and LVAD	Radiation exposure
Chest x-ray	Whole scale 2-D imaging of heart and LVAD. Usually used for rapid evaluation before further testing.	Radiation exposure, 2-D planar imaging
Magnetic resonance imaging (MRI)	Generally contraindicated in cardiac implantable devices such as LVADs	Contraindicated

### 2.1. Standard approaches

Various echocardiographic techniques with different acoustic windows are used clinically to evaluate patients with LVADs and other cardiac devices. These include the standard Transthoracic Echocardiography (TTE), Transesophageal Echocardiography (TEE), and the more recently described Transhepatic Echocardiography (THE) (10). Echocardiography offers rapid and accessible assessment of the LVAD, making it a mainstay for surveillance and troubleshooting the device. Concomitantly, right heart heart catheterization is routinely performed in patients with LVADs as a primary method to ensure adequate unloading. Hemodynamic ramp testing with concomitant echocardiography and right heart catheterization is standard in most centers. Ramp testing has evolved in recent years to become a clinically useful method to optimize pump speed, and ensure adequate unloading (8, 9). Adequately unloading using the RAMP studies is associated with a decrease in HRAEs, but the invasiveness of right heart catheterization is a significant drawback (11, 12). An alternative method, known as echocardiographic ramp testing, utilizes echocardiography without right heart catheterization. This method is simpler and less invasive, and has been validated to support pump speed changes and reduce adverse events in patients with HM3 LVADs (12). However, before this technique can be independently relied upon, further advances in echocardiographic flow and pressure analysis are needed.

TTE is the standard echocardiographic view used regularly in cardiac care. With TTE in LVAD patients, artifact is often encountered which inhibits a clinician's ability to accurately asses the device and heart. A newer, widely used echocardiographic method is TEE. TEE visualizes the structures of the heart from inside the esophagus, posterior to the device, allowing the echo beam to bypass the area of the chest where the device sits. It is frequently used during LVAD implantation to determine appropriate device orientation (13). TEE can provide a more clear view of the atria and valves than TTE, even within non-LVAD patients. TEE is more sensitive than TTE and can provide valuable information regarding hemodynamics (14). While TEE can allow for improved visualization, it is an invasive procedure and requires sedation. In addition to its invasive nature, TEE's requirement of sedation means this technique cannot be used effectively in ramp testing as sedation drugs alter hemodynamics, diminishing the value of any pump speed based hemodynamic data acquired this way.

Radiographic imaging techniques, although less commonly used, may be helpful in obtaining detailed views of the LVAD. In LVAD patients, computed tomography (CT) is particularly useful in evaluating inflow and outflow position, orientation relative to the left ventricle and aorta, and suspected complications such as outflow thrombus or twist or infection related complications such as pseudoanuerysm (15). Outflow twist is a significant complication and was of particular interest in the first iteration of the HM3 before the FDA issued a warning (16). CT is an effective tool to diagnose outflow related issues. However, since outflow twist can be dynamic process, a combined fluoroscopic angiography and CT were shown to be useful (17). The use of cardiac CT in general is minimized due to the radiation exposure, but it remains a valuable tool for acquiring high quality images when echocardiography falls short. CT images have limitations due to artifact and hyperlucency from implanted devices, which impair visualization of important structures (18). However, there are newer artifact reduction algorithm that may help circumvent these issues (19). Another technology that is important for assessment of LVAD related infections is cardiac positron emission tomography (PET). Cardiac PET has proven to be a reliable tool for diagnosing infection in LVAD patients (7).

Magnetic resonance imaging (MRI), though an extremely useful imaging technology in other contexts, is contraindicated in LVAD patients, because all relevant pumps are made with ferromagnetic materials. Fully overcoming this challenge and enabling use of MRI in LVAD patients is dependent upon innovation of pump design to incorporate only non-magnetic materials. Currently, this seems unlikely, considering that the most advanced VADs produce flow using electromagnetically levitated components to avoid damaging the blood. However, MRI can be powerful prior to LVAD implantation in order to produce highly accurate volumetric images of patients' cardiac anatomy for use in surgical planning. MRI produces high quality digital renditions of patient anatomy, and models produced from processing that data would be highly accurate. Additionally, flow through non-ferrous experimental models can be studied at high resolution via MRI, which will be discussed in a later section of this manuscript.

### 2.2. Novel approaches

Where current standard imaging techniques fall short in their ability to provide sufficient assessment of LVAD patients, novel methods have been developed in an attempt to overcome these limitations. These methods include both combinations and adaptations of typical approaches.

**Figure 1:**
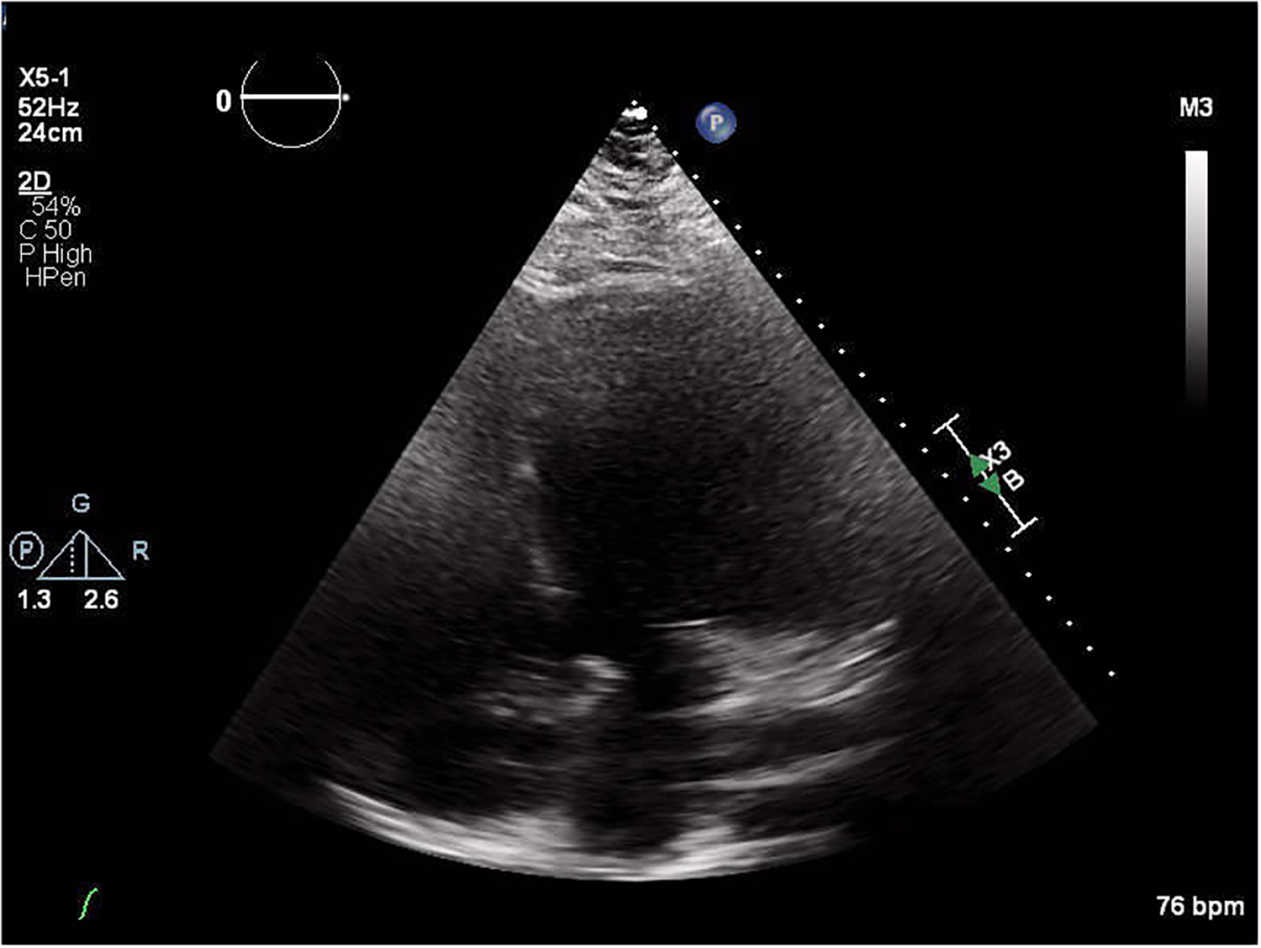
Echocardiograph of an LVAD patient depicting the result of poor acoustic windows.

For example, echocardiography has been adapted in a number of ways. Echocardiography is one of the most highly utilized techniques in assessing cardiovascular health due to its ease of use and non-invasive nature (20). Despite this, its effectiveness in LVAD patients is still limited by the appearance of artifact on the images. Artifact caused by the metal components of the device can block views of essential cardiac structures from the typical TTE view, diminishing diagnostic power, especially in patients whose LVAD positioning results in particularly poor acoustic windows (Figure 1). Some alternative echo views have been introduced to overcome artifact issues.

Another relatively new echocardiographic view used for LVAD patients with poor acoustic windows that maintains a non-invasive approach is the transhepatic (THE) view. In THE, the echo probe is aimed at one of the right intercostal spaces and through the liver to achieve a 4-chamber view, avoiding interfering structures. In a 15 subject study of LVAD patients who had poor TTEs, left and right ventricular function and inflow cannula flow of LVAD patients were all successfully assessed more often with THE than TTE (21). Similar to TTE, this view is non-invasive and can be performed at the bedside.

Lastly, intracardiac echocardiography (ICE) can be performed to visualize parts of the heart that are normally obstructed from view by devices (22). This procedure is similar to right heart catheterization in vascular route and does not require sedation and thus can be valuable for assessing hemodynamics. In a case study by El Banayosy et al., an LVAD patient experiencing ventricular tachycardia and suction events underwent TTE and TEE but the cause could not be determined until ICE was performed (23).

The ease of use of these techniques varies and their usefulness is dependent on each patient's particular anatomy, but all of these have the potential to expand diagnostic capabilities for LVAD patients with patients with poor acoustic windows.

**Figure 2:**
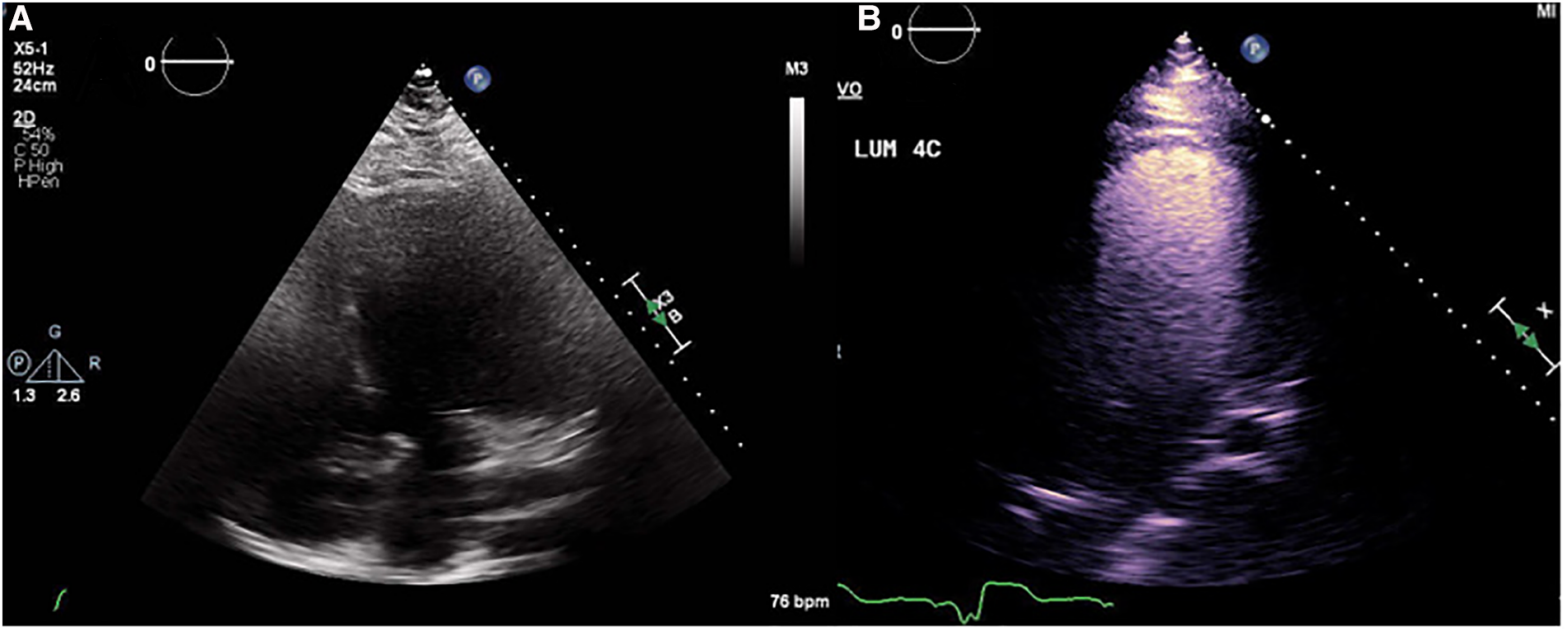
(**A**) Non contrast echocardiograph beside (**B**) contrast echocardiograph depicting the improvement in structure visualization.

Echocardiograms with poor clarity are regularly repeated and enhanced using a contrast agent in non-LVAD patients. Contrast echocardiography (CE) enhances the heart imaging capabilities by increasing opacity of walls (24). A major limitation of this technique is that contrast agents are fragile and sensitive to damage from turbulent flow such as that found in LVADs. This means their usefulness in LVAD patients is partially diminished, especially at higher pump speeds. A study conducted by Platts et al. showed that when echocardiograms were performed with contrast in LVAD patients, contrast signal intensity has an inverse relationship with pump speed (25). Despite this, use of contrast agent still improves visualization of structures in LVAD patients to an extent that treatment can be adjusted. They have been tested in LVAD patients and were proven to be safe and have no measurable effect on pump function (Figure 2) (26).

**Figure 3:**
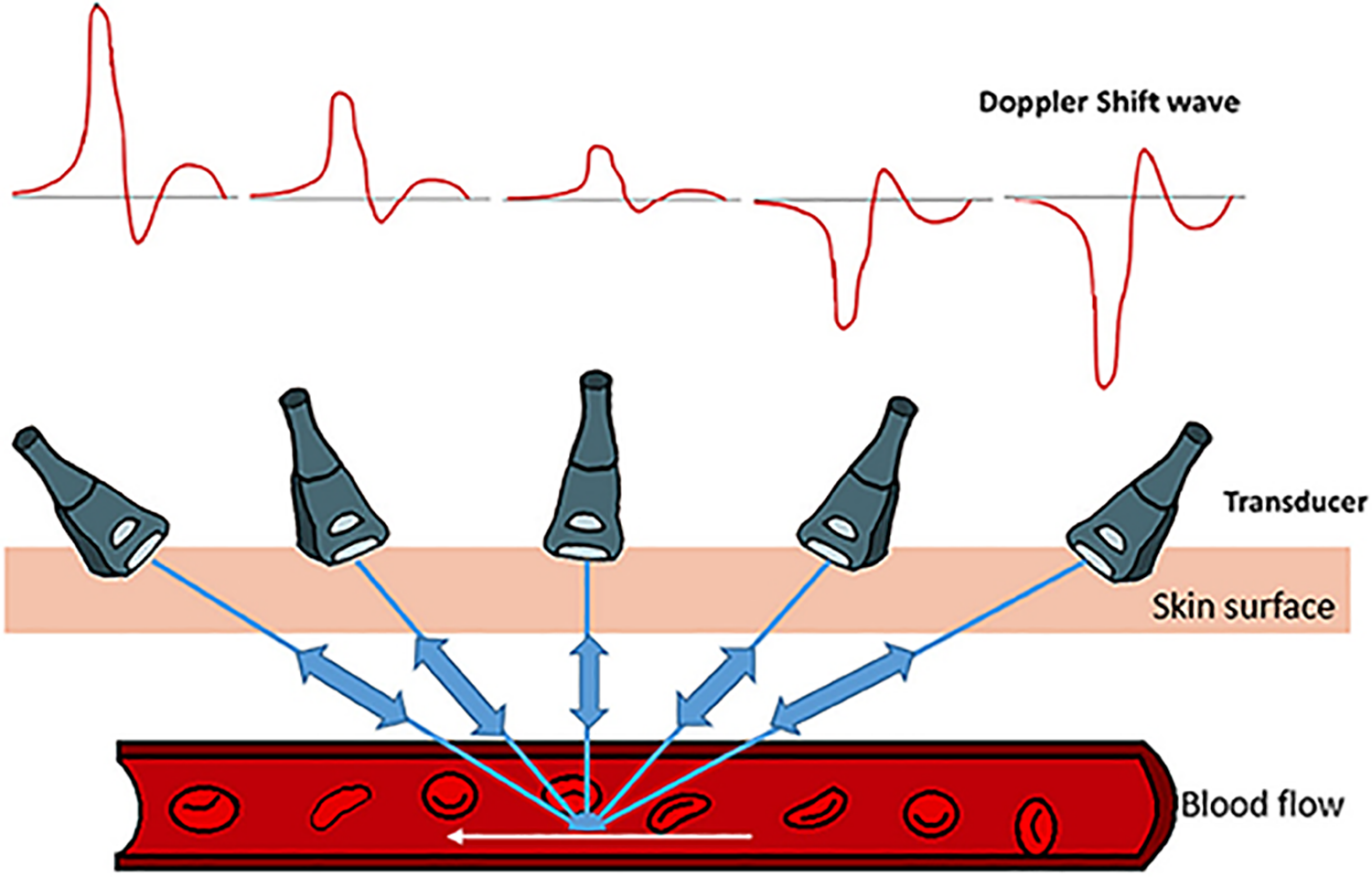
Model of Doppler echocardiograph paired with the Doppler shift wave showing how the technique assesses flow. The figure used in this work is adapted from Benslimane et al. (2019, Figure 2), with permission from the copyright holder (27).

CE can improve views, but the structure of the heart and the orientation of an LVAD within it are not the only parameters that need to be assessed. Understanding cardiac flow is essential to assessing LVAD patient health. Doppler echocardiography can be used to assess flow patterns in inflow and outflow cannula of the LVAD, however, poor acoustic windows and inability to align properly with the cannulae can limit its utility (Figure 3) (28).

Although the aforementioned imaging techniques allow for patient specific clinical monitoring, information regarding fluid dynamics such as hemodynamic flow profiles, thrombogenic potential, and fluid shear stress is currently not possible. This kind of data is more available using benchtop approaches which would be discussed in the following sections.

## 3. Benchtop evaluation of general LVAD performance

There are several techniques that have advanced in benchtop environments for potential clinical application in assessing hemodynamics of implanted devices. In general, these techniques can be categorized into two types: physical and digital (computational/numerical). These techniques are often inextricably linked in the context of modeling hemodynamic behaviors.

**Figure 4:**
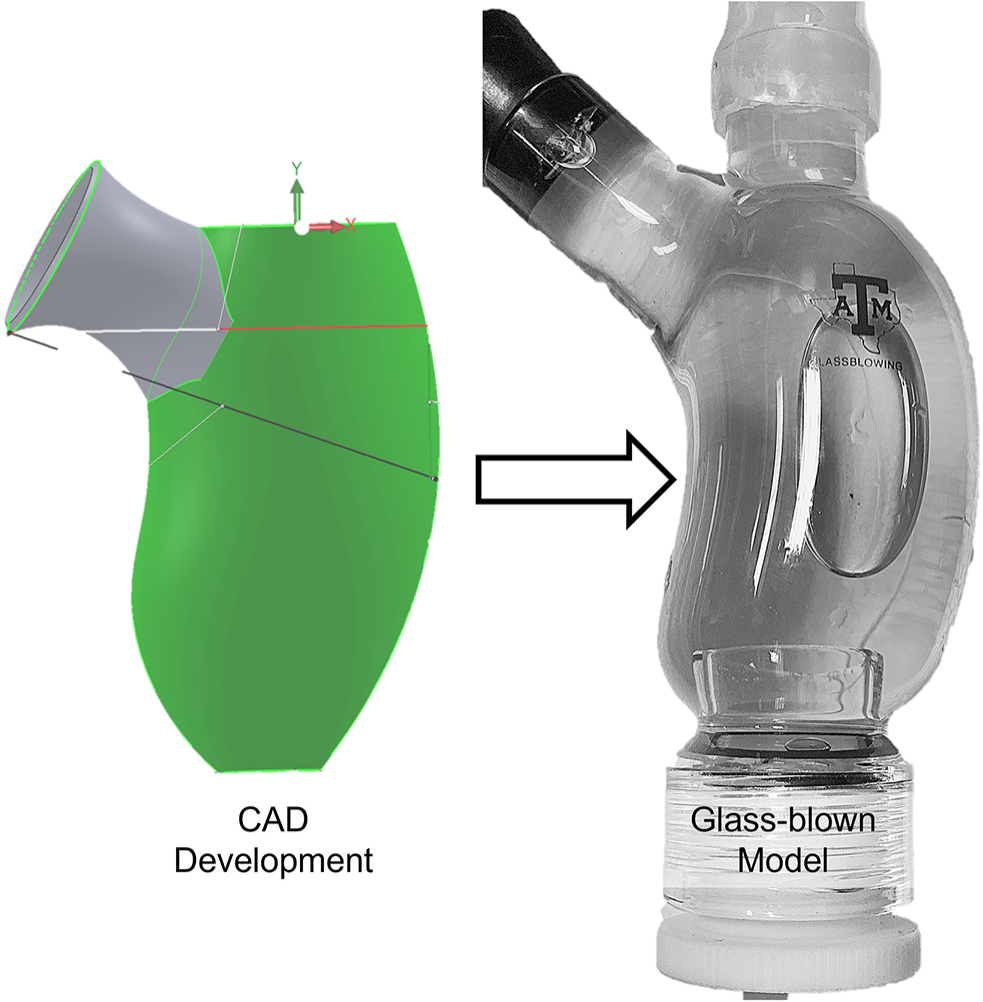
Simplified digital left ventricle model developed in CAD software and used to produce a physical, glass-blown model. This approach of developing a physical model from a computational model enables experimental validation of computational simulations.

**Figure 5:**
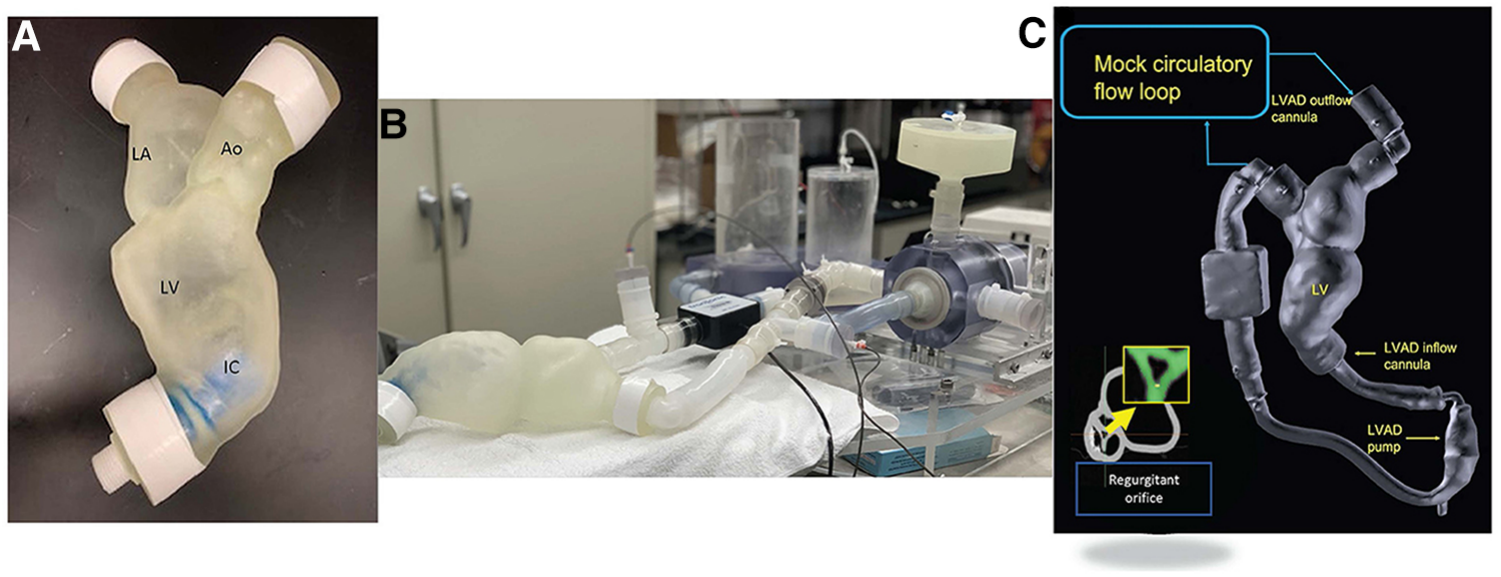
(**A**) 3d printed model of patient anatomy (atria and left ventricle), reconstructed from CT data, (**B,C**) model implemented in a mock circulatory loop, enabling study of changes in the system due to changes in VAD settings or other parameters. This set up can also be used to validate a computational model involving the same anatomy and boundary conditions (29).

Regardless of which technique is chosen, benchtop evaluation starts with creation of an anatomical model. The simplest models are useful for validation of more complex models, or to study general trends across a population due to the removal of patient-specific features. Thus, with current developments, the simpler models inform changes in treatment for populations and, once validated, allow more complex models to inform patient-specific treatment decisions. Digitally, these can be produced using computer aided design (CAD) software. The digital file can then be used to produce a physical model using many approaches, such as glass blowing (Figure 4), 3D printing, or molding. More complex models can be used to simulate patient-specific features. Digital production involves the use of imaging technologies such as CT and physical models and can subsequently be developed via 3D printing (Figure 5) or molding.

### 3.1. Physical models

Physical models are utilized more in the context of patient-specific procedural planning, patient education and surgical training/practice. Interventional cardiologists practice deployment of stents, artificial valves, left atrial appendage closure devices, and others to determine whether there may be any unexpected challenges in the actual procedure. Three dimensional models can also allow planning of device placement or patient specific identification of critical anatomical landmarks. These models can also be paired with other technologies, such as image guidance equipment, to prepare tools or guides for the operating room to ensure that the procedure is performed a certain way. Image guidance and augmented reality (AR) technologies have made their way into the operating room in the context of neurological as well as orthopedic procedures (30, 31). Similar approaches are being explored in the LVAD space, but none have made it past the benchtop yet (32).

### 3.2. Mock circulatory loops

In the context of LVADs, modeling is perhaps most useful in visualizing and simulating how an individual patient's specific hemodynamic risk factors could affect their outcomes. This involves relatively complex digital modeling, which often must be validated using physical models. This is often done using mock circulatory loops (MCL) (11, 23, 33–39). Particle image velocimetry (PIV) is also commonly combined with MCLs and physical models to study cardiac anatomy at the benchtop (20, 27, 33–45).

Mock circulatory loops have been successfully used in the context of studying valvular behavior, large vessel behavior, cardiac chamber behavior, and the performance of implanted devices (11, 21, 46–48). These MCLs often utilize 3D printed or molded anatomical models or “phantoms” to obtain more accurate results (49). In a study involving an LVAD patient with suspected aortic regurgitation (AR), an echo compatible MCL was successfully used to assess the regurgitant volume, demonstrating the usefulness of MCLs in diagnostics (50).

### 3.3. Flow visualization for validation

**Figure 6:**
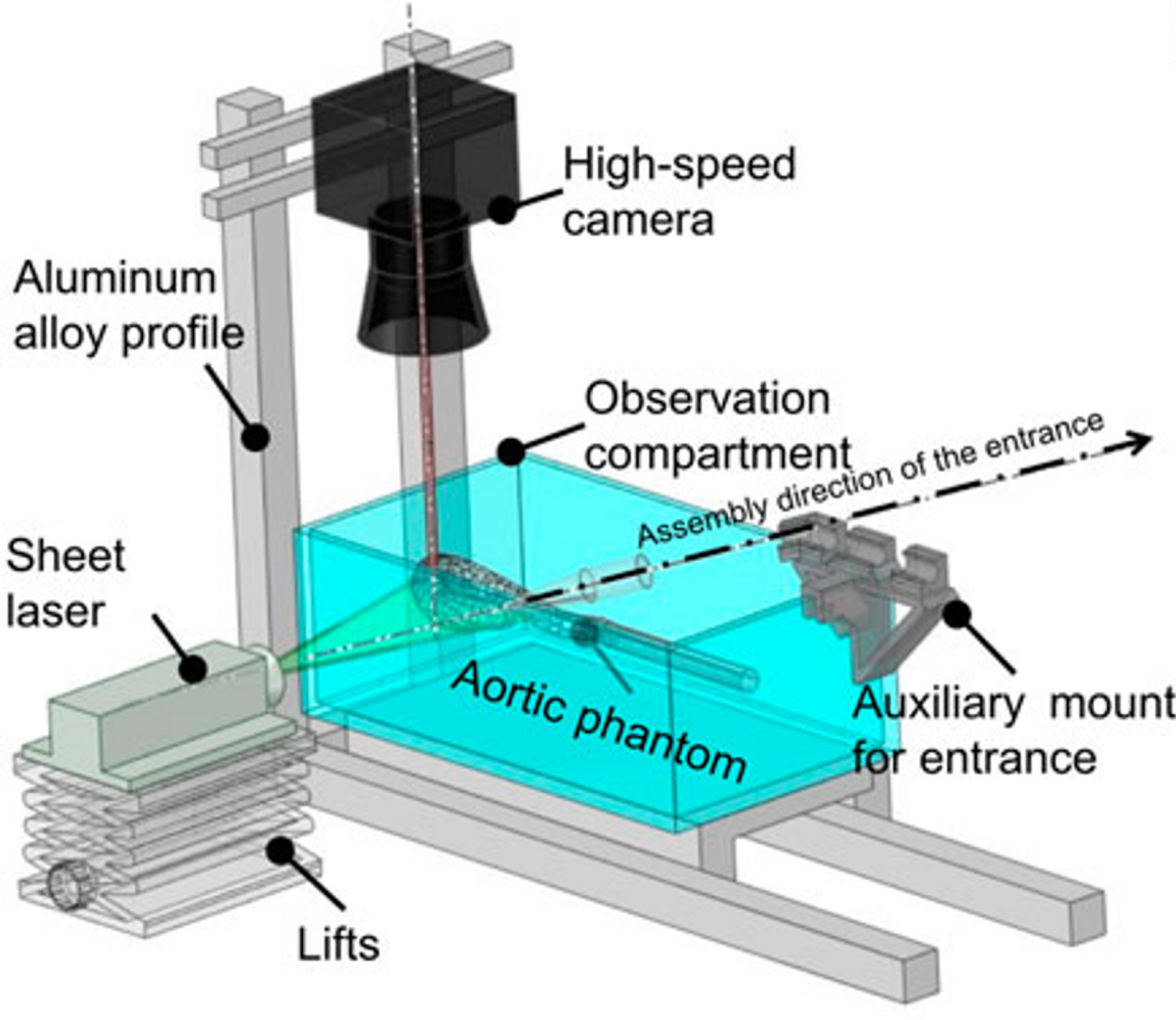
Schematic of a PIV set up for quantifying flow in an aortic model. This set up involves an optically clear anatomical model in a mock circulatory loop. Particles are introduced to the flow and illuminated in a plane using the laser sheet, and their motion is tracked and quantified using a high speed camera. The streamlines that are produced can be compared directly to those simulated via CFD. The figure used in this work is adapted from Chen et al. (2023, Figure 5), with permission from the copyright holder (51).

In order to actually obtain the data necessary for comparison to and validation of digital modeling, flow movement has to be measured and quantified. While rudimentary methods such as dye injection can be sufficient in some cases, particle image velocimetry has emerged as one of the more sophisticated methods of flow quantification. PIV requires the use of an optically clear physical model in an MCL. Small particles are released into the flow, illuminated with a planar laser, and tracked using a high-speed camera (Figure 6). Analysis of this data can be used to quantify the flow at a high resolution, and this analysis can be compared to what is produced via computational fluid dynamics simulations. This approach has been used to successfully analyze elements of fluid dynamics such as stasis and laminar vs. turbulent flow (34, 40, 42). This is a promising benchtop method because of its potential to be applied to hemodynamic adverse events in the settings of LVADs.

Another sophisticated method of flow quantification in physical models is 4D MRI. MRI compatible MCLs have been produced which enable insertion of the model into the bore of an MRI and tracking of water through the model at a very high resolution (29). The limitation of this method is its burdensome requirement for MRI compatibility, which restricts equipment such as pumps and sensors from being present in the physical model. It can be very complex to produce an MCL that sufficiently reproduces physiological flow within the physical model while maintaining MRI compatibility. Attempts to use 4D MRI for flow validation can be cumbersome due to the various translational expertise that are necessary—knowledge of experimental fluid dynamics to produce an MCL, understanding of MRI functionality and post processing of raw data to produce usable information, and also usually advanced familiarity with computational fluid dynamics (CFD) approaches to ensure that critical assumptions are being addressed and that the MCL is producing relevant conditions within the physical model. This usually implies that a heavily translational collaborative team is required to successfully use this approach.

Thus far, emphasis has been placed on the use of physical models in the context of validation of digital models. This is because physical models are limited due to the stark contrast between the mechanical behavior of the materials used to produce them and that of native tissue which makes data procured during their use difficult to rely on. This issue is exacerbated when requirements for optical clarity or MRI compatibility are introduced. In some cases, materials can be used which provide a close enough approximation for clinicians to pair their use with previous experience and education as they draw useful conclusions. Developments are being made to produce models using novel materials that more closely resemble native tissue, but these models are still far from ready to be produced for widespread clinical use (52). Even once that is possible, models like this will provide, at best, an accurate representation of how specific organ tissue *generally* behaves, rather than a patient-specific representation. Validated digital approaches allow for relatively rapid analysis of a larger number of patients, maintaining patient-specific usability while also allowing the drawing of conclusions that are relevant at the population level.

### 3.4. Computational and digital models

Introducing computational analysis to the study of blood flow gives powerful access to evaluating changes in clinically related parameters (Table 2), such as endothelial cell activation potential, particle residence time, time averaged wall shear stress, and platelet activation potential. Currently computational analysisis restricted to the academic benchtop, but development of approaches that bring this technology to the bedside would allow clinicians to make decisions for their patients based on reliable predictions of patient outcomes.

**Table 2:** Clinically significant metrics that can be studied with computational and digital approaches.

Hemodynamic metrics	Association to thrombosis	Clinical significance
Time-averaged wall shear stress	Low values	Low values related to flow stasis
Oscillatory Shear index	High values	Quantifies unidirectionality or turbulence of flow
Endothelial cell thrombus activation potential	High values	High values related to low, disturbed near-wall flow conditions
Flow shear	High values	High values are related to platelet activation and Von Willebrand factor cleavage
Particle residence time	High values	High values related to particles trapped in flow regions
Platelet activation potential	High values	High values related to an increase likelihood for platelets to activate

**Figure 7:**
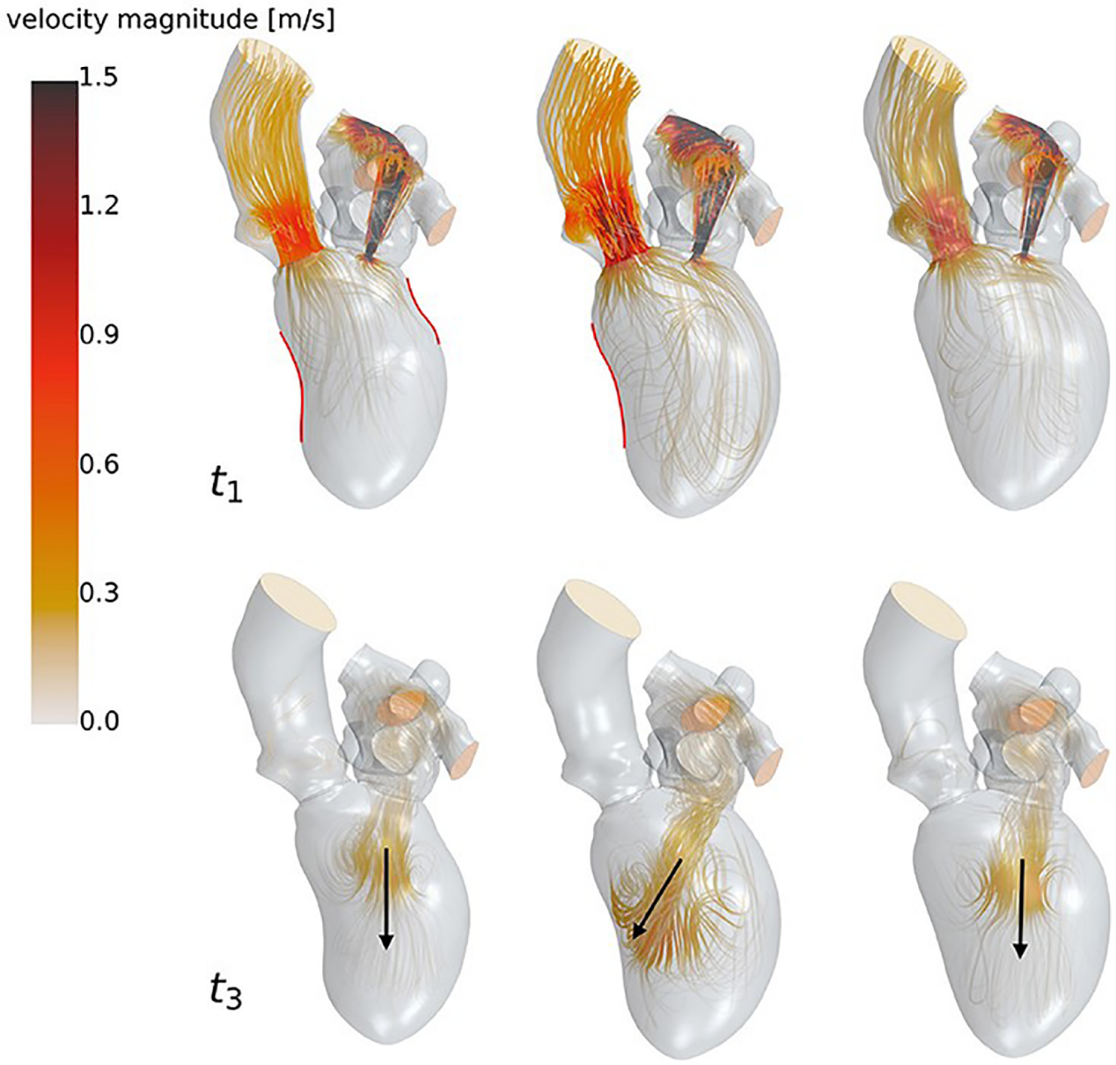
High resolution streamlines (velocity vectors) produced via CFD simulation. These are produced within CFD simulation software by using pre-set boundary conditions related to pressure and flow rate, and calculating how the particles will move from voxel to voxel within the model. Because the accuracy of these results is heavily dependent on proper methods during simulation preparation, CFD simulations must be validated experimentally before results can be considered reliable. The figure used in this work is adapted from Goubergrits et al. (2022, Figure 6), with permission from the copyright holder (53).

Computational and numerical modeling in the context of LVAD monitoring mainly involve computational fluid dynamics, which use software to mathematically and visually model blood flow through the cardiovascular system (Figure 7) (29, 35–37, 39, 54–57). Computational fluid dynamics models have been used specifically to model hemodynamic risk factors such as wall shear stress (WSS), cardiac pressures, particle residence times, and blood flow patterns in the chambers and vessels (58–61). It should be noted that computational methods such as CFD are becoming increasingly available in clinical workstations and therefore have an exciting potential to be used regularly in clinical contexts just as echocardiography is used today (60). Another interesting development in computational modeling is the translation of physical modeling to the digital space. Some studies have looked at hemodynamics of LVAD implanted hearts in a virtual mock circulatory loop model as opposed to the physical version (18, 62). As mentioned, computational modeling allows the flexibility of being able to quickly switch between different anatomies and models. One of the main benefits would be a cost perspective as software models generally cost less than physically 3D printed or manufactured models.

Despite the power digital simulations have to produce clinically relevant data, this data is not always reliable. Because these models are developed based on idealized or generalized algorithms, even the most advanced simulations cannot necessarily provide patient-specific conclusions, as they are developed using assumptions that are determined to be appropriate based on a general population (35, 44, 63). Currently, CFD has provided clinical value in vascular and valvular interventions (64–67). However, computational and numerical models used clinically in the context of mechanical circulatory support have been limited to optimization of LVAD pump speed and other parameters in order to reduce risk or severity of complications such as aortic insufficiency (35).

Rather than simulations using models from a large sample size of patients, some researchers are exploring the practicality of clinical “digital twins” (68). A digital twin is a virtual environment that is identical in all practical ways to a physical system, in which simulations can be performed to study various processes. Development of patient specific digital twins could allow simulation of device implantation, effects of pharmaceutical treatments, and other clinical interventions prior to actually performing them in a patient. However, development of patient specific virtual environments faces the same obstacles mentioned for computational and numerical simulations—it is currently far from feasible to practically quantify a patient's relevant anatomical systems and physiological behavior to a useful degree in a short enough amount of time to be useful in most cases. This challenge is exacerbated by the technical expertise required to do this work. Perhaps as artificial intelligence technology advances, clinical use of digital twins will become a more feasible prospect.

Though these mainly benchtop techniques have developed tremendously in recent years, translation to the clinical space and validation of their accuracy and utility in LVAD patients is both rare and slow.

## 4. Discussion: practical integration of benchtop approaches in the clinic

Over time, both clinical and benchtop approaches have continued to evolve, both with the development of new technologies as well as increased sophistication of standard technologies. While it is common for more sophisticated clinical approaches to have been developed in an academic/research setting, then translated from benchtop to clinic, it is rare for traditional benchtop approaches to make their way into clinical use, and there continues to be a gap between their abilities to provide clinically useful data and their accessibility by relevant clinicians. However, there are a few cases where benchtop technology has undergone translational fusion with clinical approaches and enabled novel patient evaluation in LVAD patients. The approach that embodies this translational pathway is Echocardiographic Particle Image Velocimetry (Echo PIV).

**Figure 8:**
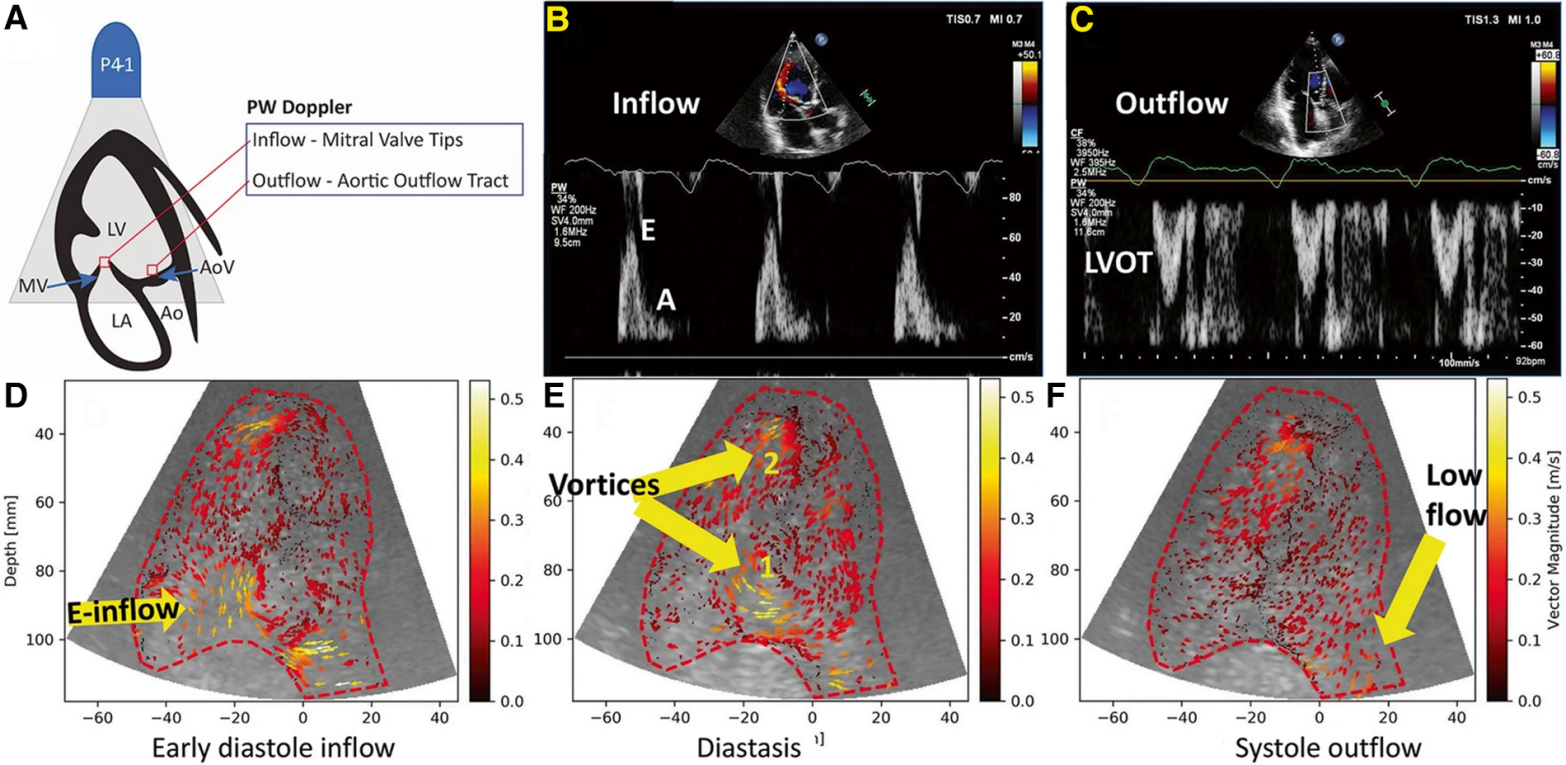
(**A**) Positioning of the Doppler and echo PIV interrogation points. (**B,C**) PW Doppler of inflow and outflow for a heart failure patient. (**D–F**) Results from echo PIV tracking flow in the same patient. This figure is adapted from Strachinaru et al. (2022, Figure 2), under a Creative Commons Attribution 4.0 International License (45).

Echo PIV (Figure 8) is the combination of echocardiography with digital particle image velocimetry analysis to create 2D velocity field measurements of cardiac flow. This technique was explored in numerous studies using controlled models, animal models and human subjects and successfully assessed cardiac function (45, 61, 69, 70). This technique was then evaluated for use in LVAD patients in a 17 subject study that showed it was safe for use in LVAD patients, did not disrupt pump function and revealed new information on the vortices formed in the left ventricle during LVAD support (71).

There have been some concerns regarding the accuracy of Echo PIV results, as it can underestimate flow velocities due to the restriction of echocardiography frame rate capture (47). New advancements seek to overcome this issue with technology such as high frame rate echocardiography that capture thousands of frames per second and particle tracking velocimetry techniques (72, 73). Other explorations attempt to make echo PIV more accessible by developing analysis software that utilize DICOM images instead of JPEGs so they are more immediately compatible with the technology used in clinical environments (46, 54).

**Figure 9:**
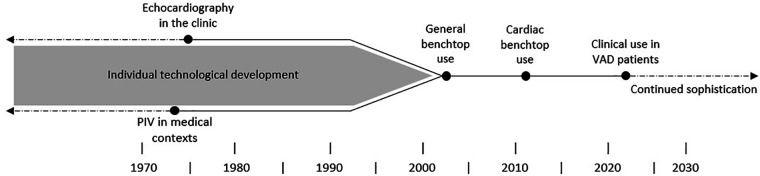
The timeline depicting the progression of echocardiography, PIV and their eventual unification in research settings.

As echo PIV is still being developed, its pathway (Figure 9) from two separate benchtop and clinical approaches to a joint approach used in patients should serve as a template to encourage translational collaborations (28, 70, 71, 74, 75). With progress like this, the analytical power of benchtop technologies can be fused with patient specific data acquisition in the clinic and brings us closer to fully informing clinical decisions, reducing complications, and improving outcomes for heart failure patients.

## References

[1] Roger VL. Epidemiology of heart failure. Circ Res. (2021) 128(10):1421–34. 10.1161/CIRCRESAHA.121.318172 33983838

[2] 2022 AHA/ACC/HFSA Guideline for the management of heart failure: a report of the American College of Cardiology/American Heart Association Joint Committee on clinical practice guidelines | Circulation. Available at: https://www.ahajournals.org/doi/10.1161/CIR.0000000000001063 (Accessed May 23, 2023). 10.1161/CIR.0000000000001063 35363499

[3] Uriel N Colombo PC Cleveland JC Long JW Salerno C Goldstein DJ Hemocompatibility-related outcomes in the MOMENTUM 3 trial at 6 months. Circulation. (2017) 135(21):2003–12. 10.1161/CIRCULATIONAHA.117.028303 28385948

[4] Varshney AS DeFilippis EM Cowger JA Netuka I Pinney SP Givertz MM. Trends and outcomes of left ventricular assist device therapy: JACC focus seminar. J Am Coll Cardiol. (2022) 79(11):1092–107. 10.1016/j.jacc.2022.01.017 35300822

[5] Mehra MR Goldstein DJ Cleveland JC Cowger JA Hall S Salerno CT Five-year outcomes in patients with fully magnetically levitated vs axial-flow left ventricular assist devices in the MOMENTUM 3 randomized trial. JAMA. (2022) 328(12):1233–42. 10.1001/jama.2022.16197 36074476 PMC9459909

[6] Allen SR Slaughter MS Ahmed MM Bartoli CR Dhingra R Egnaczyk GF COMPETENCE Trial: the EVAHEART 2 continuous flow left ventricular assist device. J Heart Lung Transplant. (2023) 42(1):33–9. 10.1016/j.healun.2022.10.011 36347767

[7] Tam MC Patel VN Weinberg RL Hulten EA Aaronson KD Pagani FD Diagnostic accuracy of FDG PET/CT in suspected LVAD infections: a case series, systematic review, and meta-analysis. JACC Cardiovasc Imaging. (2020) 13(5):1191–202. 10.1016/j.jcmg.2019.04.024 31326483 PMC6980257

[8] Kasinpila P Kong S Fong R Shad R Kaiser AD Marsden AL Use of patient-specific computational models for optimization of aortic insufficiency after implantation of left ventricular assist device. J Thorac Cardiovasc Surg. (2021) 162(5):1556–63. 10.1016/j.jtcvs.2020.04.164 32653292 PMC7666659

[9] Lim HS Hsich E Shah KB. International society of heart and lung transplantation position statement on the role of right heart catheterization in the management of heart transplant recipients. J Heart Lung Transplant. (2019) 38(3):235–8. 10.1016/j.healun.2018.12.009 30638836 PMC6816339

[10] Lim HS Howell N Ranasinghe A. The physiology of continuous-flow left ventricular assist devices. J Card Fail. (2017) 23(2):169–80. 10.1016/j.cardfail.2016.10.015 27989869

[11] Uriel N Burkhoff D Rich JD Drakos SG Teuteberg JJ Imamura T Impact of hemodynamic ramp test-guided HVAD speed and medication adjustments on clinical outcomes. Circ Heart Fail. (2019) 12(4):e006067. 10.1161/CIRCHEARTFAILURE.119.006067 30946600

[12] Najjar E Thorvaldsen T Dalén M Svenarud P Kristensen AH Eriksson MJ Validation of non-invasive ramp testing for HeartMate 3. ESC Heart Fail. (2020) 7(2):663–72. 10.1002/ehf2.12638 32037731 PMC7160500

[13] Chumnanvej S Wood MJ MacGillivray TE Melo MFV. Perioperative echocardiographic examination for ventricular assist device implantation. Anesth Analg. (2007) 105(3):583. 10.1213/01.ane.0000278088.22952.82 17717209

[14] O’Rourke MC Goldstein S Mendenhall BR. Transesophageal echocardiogram. In: Statpearls. Treasure Island: StatPearls Publishing (2023) 3. Available at: http://www.ncbi.nlm.nih.gov/books/NBK442026/ (Accessed May 22, 2023). 28723055

[15] Yuzefpolskaya M Ladanyi A Bokhari S Jorde UP Colombo PC. Effect of left ventricular unloading by pump speed adjustment on myocardial flow in continuous-flow left ventricular assist device patients. ASAIO J. (2022) 69(5):460–6. 10.1097/MAT.0000000000001875 36516021

[16] Konukoğlu O Mansuroğlu D Yıldırım Ö Bakshaliyev S Sever K Balkanay M. Outflow graft twisting of heartmate III left ventricular-assisted device: a case report. Turk Gogus Kalp Damar Cerrahisi Derg. (2019) 27(4):568–71. 10.5606/tgkdc.dergisi.2019.17871 32082927 PMC7018150

[17] Wamala I Kneissler S Kaufmann F Eulert-Grehn JJ Potapov E Dreysse S Computed tomography and fluoroscopic angiography in management of left ventricular assist device outflow graft obstruction. JACC: Cardiovasc Imaging. (2020) 13(9):2036–42. 10.1016/j.jcmg.2019.11.018 31954648

[18] Shroff GS Ocazionez D Akkanti B Vargas D Garza A Gupta P CT Imaging of complications associated with continuous-flow left ventricular assist devices (LVADs). Semin Ultrasound CT MR. (2017) 38(6):616–28. 10.1053/j.sult.2017.07.005 29179901

[19] Aissa J Boos J Sawicki LM Niklas H Karl K Martin S Iterative metal artefact reduction (MAR) in postsurgical chest CT: comparison of three iMAR-algorithms*.* Br J Radiol*.* 2017;90(1079):20160778. 10.1259/bjr.20160778 28830194 PMC5963368

[20] Estep JD Stainback RF Little SH Torre G Zoghbi WA. The role of echocardiography and other imaging modalities in patients with left ventricular assist devices. JACC Cardiovasc Imaging. (2010) 3(10):1049–64. 10.1016/j.jcmg.2010.07.012 20947051

[21] Strachinaru M Bowen DJ Constatinescu A Manintveld OC Brugts JJ Geleijnse ML Transhepatic echocardiography: a novel approach for imaging in left ventricle assist device patients with difficult acoustic windows. Eur Heart J Cardiovasc Imaging. (2020) 21(5):491–7. 10.1093/ehjci/jeaa002 32025715 PMC7167747

[22] Kassi M Rosenbaum AN Wiley BM Behfar A. Novel use for intracardiac echocardiography: evaluation of patients with continuous flow left ventricular assist devices. JACC Cardiovasc Imaging. (2019) 12(2):363–6. 10.1016/j.jcmg.2018.06.022 30219402

[23] Banayosy AE Koerner MM Brehm C Stevenson ER Pae WE Clemson B Intracardiac echocardiography for diagnosis and management of left ventricular assist device inlet obstruction. ASAIO J. (2014) 60(6):e1. 10.1097/MAT.0000000000000142 25232772

[24] Dijkmans PA Juffermans LJM Musters RJP Wamel Av ten Cate van Gilst W Microbubbles and ultrasound: from diagnosis to therapy. Eur J Echocardiogr. (2004) 5(4):245–56. 10.1016/j.euje.2004.02.001 15219539

[25] Platts DG Bartnikowski N Gregory SD Scalia GM Fraser JF. Contrast microsphere destruction by a continuous flow ventricular assist device: an in vitro evaluation using a mock circulation loop. BioMed Res Int. (2017) 2017:e4907898. 10.1155/2017/4907898 PMC5572588 28884121

[26] Schinkel AFL Akin S Strachinaru M Muslem R Soliman OII Brugts JJ Safety and feasibility of contrast echocardiography for the evaluation of patients with HeartMate 3 left ventricular assist devices. Eur Heart J Cardiovasc Imaging. (2018) 19(6):690–3. 10.1093/ehjci/jex177 28950368

[27] Benslimane FM Alser M Zakaria ZZ Sharma A Abdelrahman HA Yalcin HC. Adaptation of a mice doppler echocardiography platform to measure Ccardiac flow velocities for embryonic chicken and adult zebrafish. Front Bioeng Biotechnol. (2019) 7:96. 10.3389/fbioe.2019.00096 31139625 PMC6527763

[28] Kim HB Hertzberg JR Shandas R. Development and validation of echo PIV. Exp Fluids. (2004) 36(3):455–62. 10.1007/s00348-003-0743-5

[29] Kassi M Filippini S Avenatti E Xu S El-Tallawi KC Angulo CI Patient-specific, echocardiography compatible flow loop model of aortic valve regurgitation in the setting of a mechanical assist device. Front Cardiovasc Med. (2023) 10:994431. 10.3389/fcvm.2023.994431 36844719 PMC9945256

[30] Molina CA Theodore N Ahmed AK Westbroek EM. Augmented reality-assisted pedicle screw insertion: a cadaveric proof-of-concept study. J Neurosurg Spine. (2019) 31(1):139–46. 10.3171/2018.12.SPINE181142 30925479

[31] Matthews JH Shields JS. The clinical application of augmented reality in orthopaedics: where do we stand? Curr Rev Musculoskelet Med. (2021) 14(5):316–9. 10.1007/s12178-021-09713-8 34581989 PMC8497656

[32] Rad AA Vardanyan R Lopuszko A Alt C Stoffels I Schmack B Virtual and augmented reality in cardiac surgery. Braz J Cardiovasc Surg. (2022) 37(1):123–7. 10.21470/1678-9741-2020-0511 34236814 PMC8973146

[33] Bardi F Gasparotti E Vignali E Avril S Celi S. A hybrid mock circulatory loop for fluid dynamic characterization of 3D anatomical phantoms. IEEE Trans Biomed Eng. (2023) 70(5):1651–61. 10.1109/TBME.2022.3224581 36423318

[34] Borchers P Walter M Leonhardt S Telyshev D Pugovkin A. Flow profile generation for a left ventricular assist device using iterative learning control. Curr Dir Biomed. (2021) 7:279–82. 10.1515/cdbme-2021-2071

[35] Bozkurt S. Pressure, flow rate and operating speed characteristics of a continuous flow left ventricular assist device during varying speed support. IEEE Access. (2020) 8:129830–41. 10.1109/ACCESS.2020.3009264

[36] Christle JW Moneghetti KJ Duclos S Mueller S Moayedi Y Khush K Cardiopulmonary exercise testing with echocardiography to assess recovery in patients with ventricular assist devices. ASAIO J. (2021) 67(10):1134–8. 10.1097/MAT.0000000000001383 34570726

[37] Kado Y Smith WA Miyamoto T Adams J Polakowski AR Dessoffy R Use of a virtual mock loop model to evaluate a new left ventricular assist device for transapical insertion. Int J Artif Organs. (2020) 43(10):677–83. 10.1177/0391398820907104 32089074

[38] Kalogeropoulos AP Al-Anbari R Pekarek A Wittersheim K Pernetz MA Hampton A The right ventricular function after left ventricular assist device (RVF-LVAD) study: rationale and preliminary results. Eur Heart J Cardiovasc Imaging. (2016) 17(4):429–37. 10.1093/ehjci/jev162 26160395 PMC4793936

[39] Prather R Divo E Kassab A DeCampli W. Computational fluid dynamics study of cerebral thromboembolism risk in ventricular assist device patients: effects of pulsatility and thrombus origin. J Biomech Eng. (2021) 143(9):091001-1–09001-10. 10.1115/1.4050819 33843992

[40] Acharya D Singh S Tallaj JA Holman WL George JF Kirklin JK Use of gated cardiac computed tomography angiography in the assessment of left ventricular assist device dysfunction. ASAIO J. (2011) 57(1):32–7. 10.1097/MAT.0b013e3181fd3405 20966744

[41] Meyers BA Goergen CJ Segers P Vlachos PP. Colour-Doppler echocardiography flow field velocity reconstruction using a streamfunction-vorticity formulation. J R Soc Interface. (2020) 17(173):20200741. 10.1098/rsif.2020.0741 33259749 PMC7811584

[42] Ghodrati M Khienwad T Maurer A Moscato F Zonta F Schima H Validation of numerically simulated ventricular flow patterns during left ventricular assist device support. Int J Artif Organs. (2021) 44(1):30–8. 10.1177/0391398820904056 32022612 PMC7780364

[43] Addetia K Kruse E Kim G Guile B Hipke K Lang RM. A new strategy for left ventricular assist device outflow graft interrogation using ultrasound contrast. J Am Soc Echocardiogr. (2021) 34(4):445–7. 10.1016/j.echo.2020.12.001 33316397

[44] Telyshev D Petukhov D Selishchev S. Numerical modeling of continuous-flow left ventricular assist device performance. Int J Artif Organs. (2019) 42(11):611–20. 10.1177/0391398819852365 31169054

[45] Singh M Malik N Brar V Bering PT Hadadi C Sheikh FH Ventricular fibrillation in a left ventricular assist device patient: can the echocardiogram be misleading? J Cardiovasc Electrophysiol. (2021) 32(3):862–6. 10.1111/jce.14904 33484203

[46] Shandas R Zhang F Chen J Mazzaro LA. Direct echo particle image velocimetry flow vector mapping on ultrasound dicom images. (2014). Available at: https://patents.google.com/patent/US20140147013A1/en (Accessed April 25, 2023).

[47] Strachinaru M Voorneveld J Keijzer LBH Bowen DJ Mutluer FO ten Cate F Left ventricular high frame rate echo-particle image velocimetry: clinical application and comparison with conventional imaging. Cardiovasc Ultrasound. (2022) 20(1):11. 10.1186/s12947-022-00283-4 35473581 PMC9040345

[48] Tang PC Duggal NM Haft JW Aaronson KD Pagani FD. Fate of preoperative moderate mitral regurgitation following left ventricular assist device implantation. J Card Surg. (2021) 36(6):1843–9. 10.1111/jocs.15428 33604994 PMC11196981

[49] Stapor M Pilat A Gackowski A Misiuda A Gorkiewicz-Kot I Kaleta M Echo-guided left ventricular assist device speed optimisation for exercise maximisation. Heart. (2022) 108(13):1055–62. 10.1136/heartjnl-2021-320495 35314453 PMC9209671

[50] Kassi M Filippini S Agrawal T Bhimaraj A Guha A Trachtenberg B Proof of concept: echo compatible mock-circulatory flow loop to accurately quantify aortic regurgitation in patients with left ventricular assist devices. J Heart Lung Transplant. (2020) 39(4, Suppl):S415. 10.1016/j.healun.2020.01.184

[51] Chen H Wang W Liu D Cao Z Yang Y He Y The effect of terminal impedance on aortic morphology and hemodynamics: an in vitro phantom study using flow field visualization. Front Bioeng Biotechnol. (2023) 11:1175916. 10.3389/fbioe.2023.1175916 37168613 PMC10165012

[52] Mirdamadi E Tashman JW Shiwarski DJ Palchesko RN Feinberg AW. FRESH 3D bioprinting a full-size model of the human heart. ACS Biomater Sci Eng. (2020) 6(11):6453–9. 10.1021/acsbiomaterials.0c01133 33449644

[53] Goubergrits L Vellguth K Obermeier L Schlief A Tautz L Bruening J CT-based analysis of left ventricular hemodynamics using statistical shape modeling and computational fluid dynamics. Front Cardiovasc Med. (2022) 9:901902. 10.3389/fcvm.2022.901902 35865389 PMC9294248

[54] DeMarchi N White C. Echo particle image velocimetry. J Vis Exp. (2012) (70):e4265. 10.3791/4265 PMC3712581 23299186

[55] Imamura T Narang N Kim G Nitta D Fujino T Nguyen A Aortic insufficiency during HeartMate 3 left ventricular assist device support. J Card Fail. (2020) 26(10):863–9. 10.1016/j.cardfail.2020.05.013 32473380

[56] Kato TS Jiang J Schulze PC Serial echocardiography using tissue Doppler and speckle tracking imaging to monitor right ventricular failure before and after left ventricular assist device surgery. JACC Heart Fail. (2013) 1(3):216–22. 10.1016/j.jchf.2013.02.005 24621873 PMC3997790

[57] Mulzer J Krastev H Hoermandinger C Merke N Alhaloush M Schoenrath F Cardiac remodeling in patients with centrifugal left ventricular assist devices assessed by serial echocardiography. Echocardiography. (2022) 39(5):667–77. 10.1111/echo.15338 35393693

[58] Romanova AN Pugovkin AA Denisov MV Ephimov IA Gusev DV Walter M Hemolytic performance in two generations of the sputnik left ventricular assist device: a combined numerical and experimental study. J Funct Biomater. (2022) 13(1):7. 10.3390/jfb13010007 35076513 PMC8788462

[59] Ruan DT Farr M Ning Y Kurlansky P Sayer G Uriel N The role of serial right heart catheterization survey in patients awaiting heart transplant on ventricular assist device. ASAIO J. (2022) 68(5):663. 10.1097/MAT.0000000000001542 34352817

[60] Callington A Long Q Mohite P Simon A Mittal TK. Computational fluid dynamic study of hemodynamic effects on aortic root blood flow of systematically varied left ventricular assist device graft anastomosis design. J Thorac Cardiovasc Surg. (2015) 150(3):696–704. 10.1016/j.jtcvs.2015.05.034 26092505

[61] Voorneveld J Keijzer LBH Strachinaru M Bowen DJ Goei JSL Cate FT High-frame-rate echo-particle image velocimetry can measure the high-velocity diastolic flow patterns. Circ Cardiovasc Imaging. (2019) 12(4):e008856. 10.1161/CIRCIMAGING.119.008856 30939921

[62] Malick A Ning Y Kurlansky PA Melehy A Yuzefpolskaya M Colombo PC Development of de novo aortic insufficiency in patients with HeartMate 3. Ann Thorac Surg. (2022) 114(2):450–6. 10.1016/j.athoracsur.2021.08.074 34624263

[63] Bozkurt S Bozkurt S. In-silico evaluation of left ventricular unloading under varying speed continuous flow left ventricular assist device support. Biocybern Biomed Eng. (2017) 37(3):373–87. 10.1016/j.bbe.2017.03.003

[64] Alemu Y Girdhar G Xenos M Sheriff J Jesty J Einav S Design optimization of a mechanical heart valve for reducing valve thrombogenicity—a case study with ATS valve. ASAIO J. (2010) 56(5):389. 10.1097/MAT.0b013e3181e65bf9 20613492

[65] Bianchi M Marom G Ghosh RP Fernandez HA Taylor JR Slepian MJ Effect of balloon-expandable transcatheter aortic valve replacement positioning: a patient-specific numerical model. Artif Organs. (2016) 40(12):E292–304. 10.1111/aor.12806 27911025 PMC5137806

[66] Xenos M Labropoulos N Rambhia S Alemu Y Einav S Tassiopoulos A Progression of abdominal aortic aneurysm towards rupture: refining clinical risk assessment using a fully coupled fluid–structure interaction method. Ann Biomed Eng. (2015) 43(1):139–53. 10.1007/s10439-014-1224-0 25527320 PMC4289023

[67] Alemu Y Bluestein D. Flow-induced platelet activation and damage accumulation in a mechanical heart valve: numerical studies. Artif Organs. (2007) 31(9):677–88. 10.1111/j.1525-1594.2007.00446.x 17725695

[68] Coorey G Figtree GA Fletcher DF Snelson VJ Vernon ST Winlaw D The health digital twin to tackle cardiovascular disease—a review of an emerging interdisciplinary field. NPJ Digit Med. (2022) 5(1):1–12. 10.1038/s41746-022-00640-7 36028526 PMC9418270

[69] Prinz C Faludi R Walker A Amzulescu M Gao H Uejima T Can echocardiographic particle image velocimetry correctly detect motion patterns as they occur in blood inside heart chambers? A validation study using moving phantoms. Cardiovasc Ultrasound. (2012) 10(1):24. 10.1186/1476-7120-10-24 22672727 PMC3439370

[70] Aigner P Schweiger M Fraser K Choi Y Lemme F Cesarovic N Ventricular flow field visualization during mechanical circulatory support in the assisted isolated beating heart. Ann Biomed Eng. (2020) 48(2):794–804. 10.1007/s10439-019-02406-x 31741229 PMC6949310

[71] Schinkel AFL Akin S Strachinaru M Muslem R Bowen D Yalcin YC Evaluation of patients with a HeartMate 3 left ventricular assist device using echocardiographic particle image velocimetry. J Ultrasound. (2020) 24(4):499–503. 10.1007/s40477-020-00533-z 33241488 PMC8572275

[72] Sampath K Harfi TT George RT Katz J. Optimized time-resolved echo particle image velocimetry– particle tracking velocimetry measurements elucidate blood flow in patients with left ventricular thrombus. J Biomech Eng. (2018) 140(4):041010-1–041010-14. 10.1115/1.4038886 29305613

[73] Toulemonde MEG Corbett R Papadopoulou V Chahal N Li Y Leow CH High frame-rate contrast echocardiography: in-human demonstration—ClinicalKey. (2018). Available at: https://www.clinicalkey.com/#!/content/playContent/1-s2.0-S1936878X17309713?returnurl=https:%2F%2Flinkinghub.elsevier.com%2Fretrieve%2Fpii%2FS1936878X17309713%3Fshowall%3Dtrue&referrer=https:%2F%2Fpubmed.ncbi.nlm.nih.gov%2F (Accessed May 22, 2023). 10.1016/j.jcmg.2017.09.011 29248652

[74] Maleki M Esmaeilzadeh M. The evolutionary development of echocardiography. Iran J Med Sci. (2012) 37(4):222–32. PMCID: PMC3565194, PMID: 23390327. 23390327 PMC3565194

[75] Tanaka T Riva C Ben-Sira B. Blood velocity measurements in human retinal vessels. Science. (1974) 186(4166):830–1. 10.1126/science.186.4166.830 4469681

